# Supra-ilioinguinal versus modified Stoppa approach in the treatment of acetabular fractures: reduction quality and early clinical results of a retrospective study

**DOI:** 10.1186/s13018-019-1428-y

**Published:** 2019-11-14

**Authors:** Sheng Yao, Kaifang Chen, Yanhui Ji, Fengzhao Zhu, Lian Zeng, Zekang Xiong, Tingfang Sun, Fan Yang, Jia Liu, Xiaodong Guo

**Affiliations:** 1 Department of Orthopaedics, Union Hospital, Tongji Medical College, Huazhong University of Science and Technology, Wuhan, China; 2 Department of Orthopaedics, The First Affiliated Hospital of Zhengzhou University, Zhengzhou, China; 3 Department of Orthopedics, Yongchuan Hospital, Chongqing Medical University, Chongqing, China; 4 Department of Radiology, Union Hospital, Tongji Medical College, Huazhong University of Science and Technology, Wuhan, China

**Keywords:** Supra-ilioinguinal approach, Modified Stoppa approach, Acetabular fractures, Quadrilateral plate

## Abstract

**Background:**

To compare the efficacy of the operative techniques, complications, reduction quality and hip functional recovery by using the supra-ilioinguinal approach and the modified Stoppa approach for the management of acetabular fractures.

**Methods:**

A consecutive cohort of 60 patients from September 2014 to October 2017 with displaced acetabular fractures involving the quadrilateral plate were treated operatively with supra-ilioinguinal approach (group A) and modified Stoppa approach (group B), respectively. There were 36 patients in group A and 24 patients in group B. The surgical details, complications, radiographic and clinical results were recorded. The quality of reduction was assessed by measuring the residual step and gap displacement of postoperative CT with a standardized digital method.

**Results:**

The complications, reduction quality (gaps and steps) and hip function recovery had no significant statistical difference in approaches. The mean operative time was shorter and the mean intraoperative haemorrhage was less in group A. There were statistical differences in the operative time (*P* = 0.025) and intraoperative haemorrhage (*P* = 0.003) between the supra-ilioinguinal approach and the modified Stoppa approach.

**Conclusion:**

Compared to the modified Stoppa approach, the supra-ilioinguinal approach provides a closer visualization to the quadrilateral plate, the operative time was shorter and the intraoperative haemorrhage was clearly less. It is at least equal to or could be a better choice to deal with complicated acetabular fractures especially involving the quadrilateral plate and the anterior one third of the iliac bone.

## Background

Acetabular fractures pose a great surgical challenge for orthopaedic trauma surgeons. Surgical management of displaced acetabular fracture has become the gold standard after the early work of Letournel [1]. The goals of treating an acetabular fracture are to restore the congruity and stability of the hip joint. Most should agree that anatomic reduction of weight bearing surface and concentric reduction of the hip joint is essential for long-term satisfactory results [2]. The type and nature of the acetabular fracture substantially influence which approach is used [1, 3].

The modified Stoppa approach (anterior intra-pelvic approach, AIP) allows for conveniently exposing the quadrilateral plate and the corona mortis (anastomosis of the external iliac vessel with obturator vessel) [4–6]; however, the rectus abdominis obstructs the surgical visualization and makes reduction and fixation at a further location. Moreover, anterior fracture with the iliac crest fracture usually must be accessed by an additional window of the ilioinguinal approach [7, 8].

The ilioinguinal approach is one of the standard approaches for the treatment of acetabular fractures [9]. With this approach, the inner side of the acetabulum, especially the quadrilateral plate cannot be visualized directly during the surgery. Furthermore, it causes great trauma to the patient and is prone to complications, such as inguinal hernia [10, 11]. A new intra-pelvic approach, the supra-ilioinguinal approach, beyond the classical ilioinguinal was introduced in order to make it easier to manage the acetabular fracture involving the quadrilateral plate [12]. Unlike another similar surgical approach, the pararectus approach [13, 14], it directly incises the rectus abdominis instead of the rectus sheath, which may reduce the occurrence of iatrogenic injury of the peritoneum and abdominal wall hernia. In addition, this approach is located more laterally, no additional incisions are needed to fix the iliac crest fracture of the high anterior column fracture.

In this retrospective study, we compared the supra-ilioinguinal approach and the modified Stoppa approach for the treatment of acetabular fractures. Our specific aims were to confirm the efficacy of the supra-ilioinguinal approach through comparing the operative time, intraoperative haemorrhage, postoperative complication rates, anatomic reduction quality and hip function recovery between the two approaches.

## Patients and methods

After institutional review board approval, a consecutive cohort of 60 patients with acetabular fractures at a level 1 trauma centre from September 2014 to October 2017 were treated operatively using supra-ilioinguinal approach (group A) and modified Stoppa approach (group B), respectively. The patients were regularly followed up for at least 1 year. The distribution of the fractures according to Letournel-Judet classification is shown in Table 1. Simple posterior wall fracture and patients who were not appropriate for surgery for any other reasons were excluded. The radiographs and medical records of these patients were retrospectively analysed.

**Table 1:** Patient demographics

	Supra-ilioinguinal (*n* = 36)	Modified Stoppa (*n* =24)	Test value	*P* value
Age (years)	43.4 (23–71, 13.6)	44.5 (23–78, 14.4)	0.314	0.754^#^
Male gender	26 (72.2%)	16 (66.7%)	0.212	0.645^*^
Mechanism of injury			0.100	0.752^*^
Traffic accident	18 (50.0%)	13 (54.2%)		
Fall from height (≧ 3 m)	18 (50.0%)	11 (41.9%)		
Fracture classification			0.670	0.955^*^
AC + AW	4 (11.1%)	3 (12.5%)		
Transverse	5 (13.9%)	3 (12.5%)		
T-type	5 (13.9%)	5 (20.8%)		
ACPHT	9 (25.0%)	6 (25.0%)		
Both column	13 (36.1%)	7 (29.2%)		
Multiple injuries			0.791	0.978^*^
Pelvic fracture	7 (19.4%)	5 (20.8%)		
Extremity fracture	7 (19.4%)	3 (12.5%)		
Spine fracture	1 (2.8%)	1 (4.2%)		
Pleural effusion	11 (30.6%)	9 (37.5%)		
Craniocerebral injury	2 (5.6%)	1 (4.2%)		
Time to surgery (days)	7.1 (3–20, 3.4)	7.2 (3–15, 3.0)	0.097	0.923^#^

Categorical variables are given as absolute numbers with percentages in parentheses. Noncategorical variables are given as mean (range, SD)

*AC* anterior column, *AW* anterior wall, *ACPHT* anterior column with posterior hemitransverse

*Pearson Chi-square test

^#^Two independent samples Student’s *t* test

Group A contained 36 (26 males and 10 females) patients with a mean age of 43.4 (range 23–71, SD 13.6) years old; open reduction and fixation was performed using the supra-ilioinguinal approach, while modified Stoppa approach was used in group B which contained 24 (16 males and 8 females) patients with a mean age of 44.5(range 23–78, SD 14.4) years old. The mechanism of injury was traffic accident and fall from height (≧3 m). Combined injuries included pelvic fracture on the contralateral side, extremity fracture, spine fracture, pleural effusion and craniocerebral injury. The fracture patterns in group A were 4 for anterior column and/or anterior wall, 5 for transverse, 5 for T-type, 9 for anterior column with posterior hemitransverse, and 13 for both columns. The fracture patterns in group B were 3 for anterior column and/or anterior wall, 3 for transverse, 5 for T-type, 6 for anterior column with posterior hemitransverse, and 7 for both columns. The mean time from injury to operation was 7.1 (range 3–20, SD 3.4) days and 7.2 (range 3–15, SD 3.0) days, respectively. Demographic data is presented in Table 1. The operative time, intraoperative haemorrhage, perioperative complications and follow-up data were documented.

### Pre-operative preparations

As soon as these patients arrived at our emergency service, adequate resuscitation and haemodynamic stability was achieved. Skeletal traction of distal femur was conventionally used, and low molecular weight heparin (10 mg, qd) was used for prevention of deep vein thrombosis. Pelvic X-ray, computerized tomography (CT) scan and three-dimensional (3D) reconstruction were routinely performed. A Doppler ultrasound of the pelvis and the lower extremities was done to rule out any deep venous thrombosis (DVT). The 3D printing technology was used in some complicated cases to print pelvic model, and the contralateral hemipelvis was also mirror-printed to preshape plates on the model. One day before the surgery, routine enema, urinary catheterization and preparations for blood transfusion must all be reassessed.

### Surgical procedures

**Fig. 1:**
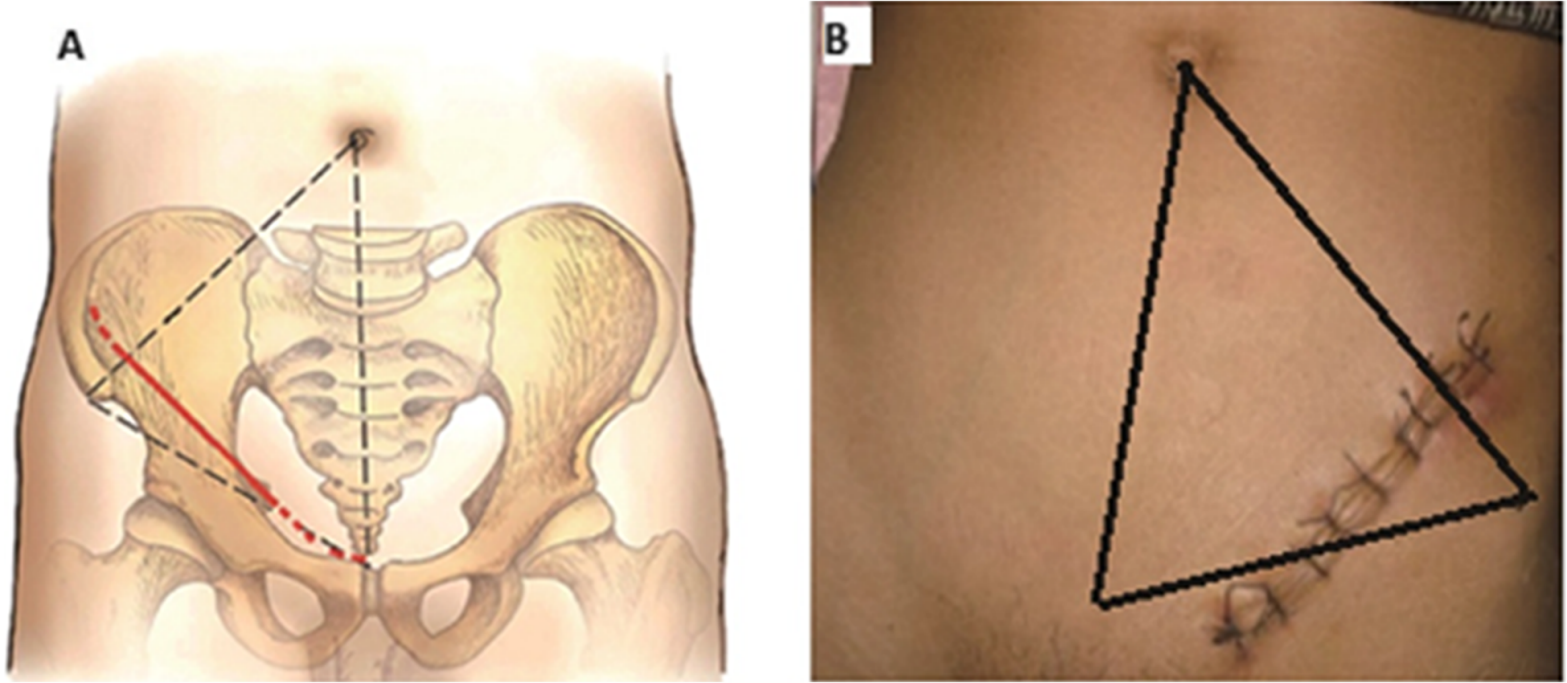
Schematic graph of the supra-ilioinguinal approach (**a**). The incision starts at the lateral one fourth of the line connecting the umbilicus with the ASIS and ends at the medial thirds of the line connecting the ASIS and the symphysis. The incision line postoperative (**b**)

**Fig. 2:**
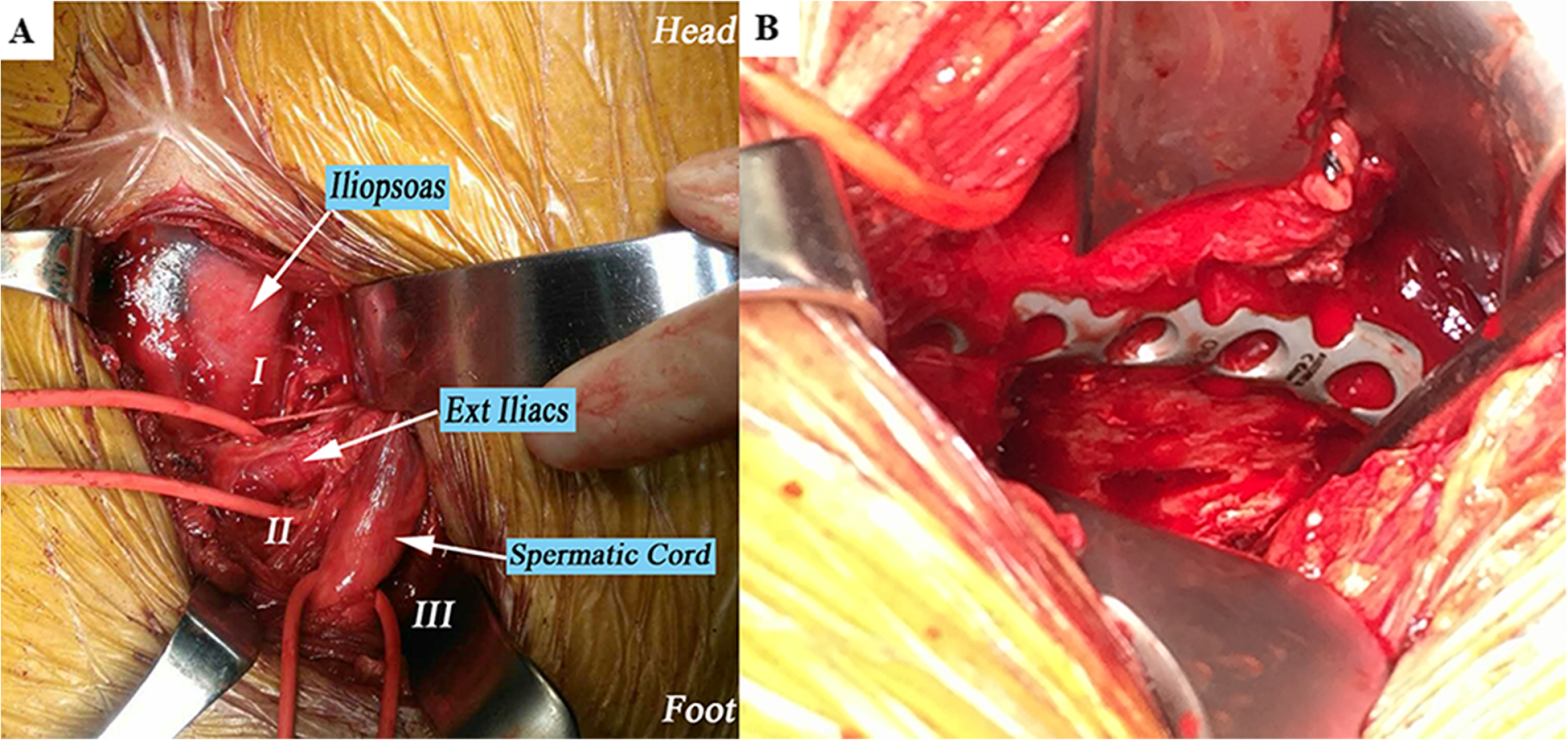
Surgical visualization of the supra-ilioinguinal approach. I, II and III were the first to the third window, respectively (**a**). An infra-pectineal plate through the medial window of the supra-ilioinguinal approach (**b**)

Patients in group A were operated using the supra-ilioinguinal approach as described in the previous article [12]. After anaesthesia, the patient was placed in a supine position on a radiolucent table, surgeon was positioned on the side contralateral to the fractured acetabulum. The umbilicus, the anterior superior iliac spine (ASIS) and the symphysis pubis are the three landmarks for the incision. The incision started at the junction of the lateral and the middle 1/4 of the line connecting the umbilicus with the ASIS, continuing in a slightly convex curve in a distal medial direction towards the junction of the middle and the medial thirds of a line connecting the ASIS with the symphysis (Fig. 1). We recommend the following three anatomical windows of this approach: (1) the first window (lateral window) is located between the ilium and the iliopsoas, (2) the second window (middle window) is located between the iliopsoas and iliac blood vessel bundle and (3) the third window (medial window) is located between the vascular bundle and the spermatic cord/round ligament. All three windows and exposed anatomical structures can be clearly seen in Fig. 2a. When symphysis diastases occurred, the incision was extended to the contralateral side. In group B, the surgical exposure in the modified Stoppa approaches was done following the steps described by Sagi et al. [6].

Supra-/infra-pectineal plate along with the pelvic brim was applied to provide the main mechanical support for acetabular fracture fixation. A comminuted quadrilateral surface was supported by a buttress plate through the medial window of the supra-ilioinguinal approach (Fig. 2b). The posterior column was commonly fixed with lag screws (Fig. 4). However, a reconstruction plate attached to the medial side of the posterior column and distal to the ischial spine was an alternative fixation method. To avoid the possibility of screws penetrating the hip joint, they were inserted according to recommendations based on the determination of safe zones or absolutely and relatively hazardous regions for screw placement [15]. No weight bearing on the operated side was recommended for all the patients for at least 1 months.

### Radiographic analysis and follow-up

Pelvic radiographs and CT scans were routinely taken within 1 week after surgeries and reviewed by an independent orthopaedic trauma surgeon. CT images were obtained with 1–2-mm slice thickness, multiplanar and 3D reconstruction were reconstructed using a standardized protocol. The assessment of reduction of the fractures postoperatively was graded according to the Matta criteria [2]: anatomical (0–1 mm), imperfect (2–3 mm) or poor (> 3 mm).

**Fig. 3:**
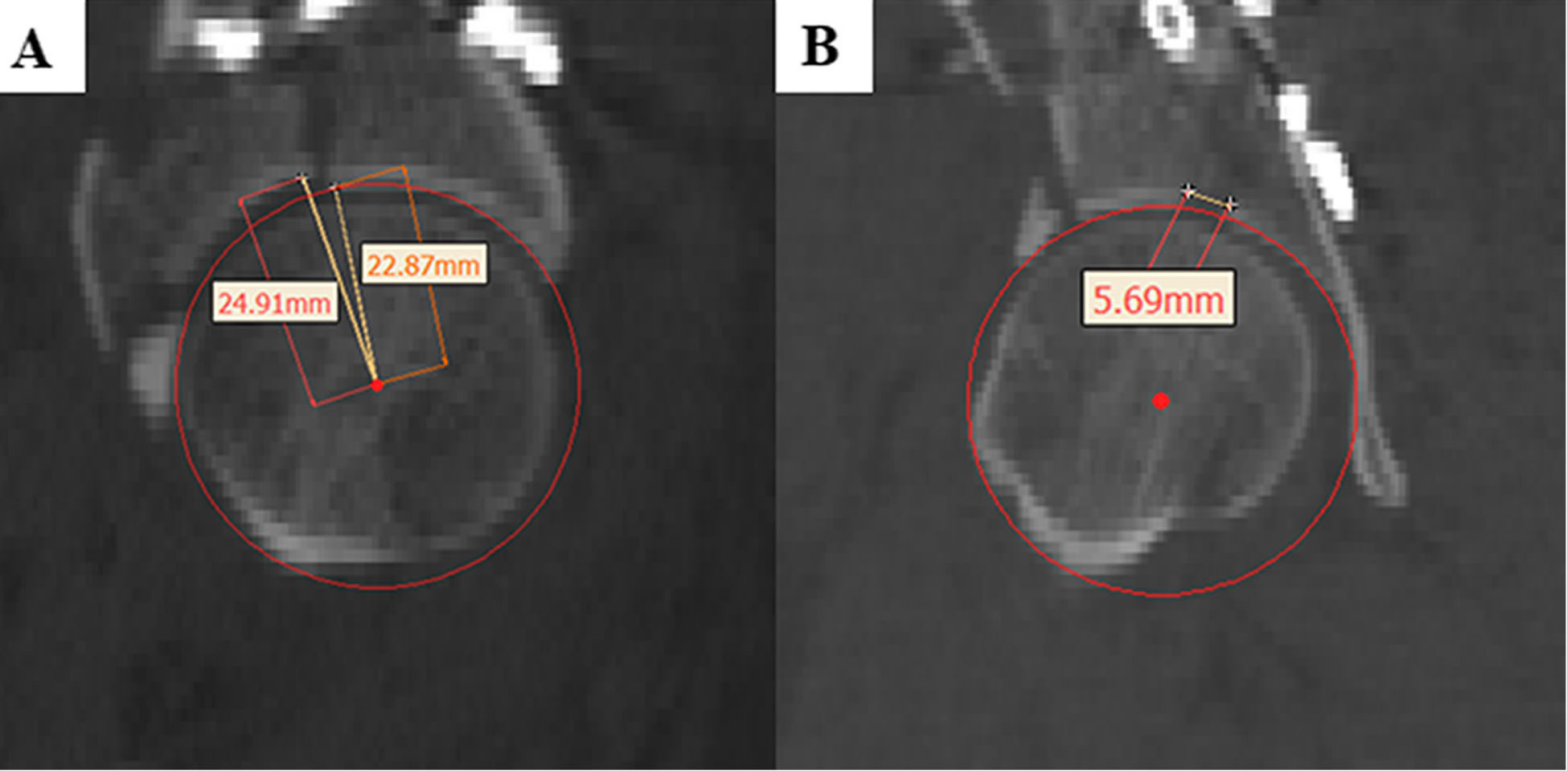
The measurement of residual displacement. The measurement of step (of 2.04 mm) is obtained by the difference between the distance from the most displaced fragment to the centre (of 24.91 mm) of the circle template and the radius of the circle template (of 22.87 mm) (**a**). The gap displacement (of 5.69 mm) is measured in-line with a digital circular template created to match the intact (or largest) portion of the acetabular dome (**b**)

A standardized digital CT-based method [16] was used to measure residual step and gap displacement of postoperative CT. According to the procedure, the greatest gap or step displacement in any of the axial, sagittal or coronal plane views was measured only in the 1-cm area of weight-bearing dome. The measurement of the step was obtained by the difference between the two measurements, including the distance from the subchondral bone of the most displaced fragment to the centre of the circle template and the radius of the circle template (Fig. 3a).The residual gap was measured in-line with the acetabular dome, with a digital circular template created to match the intact (or largest) portion of the area (Fig. 3b). All measurement work was done in Mimics 19.0 (Materialise, Leuven, Belgium) software. The greatest intra-articular gap and step displacement detected in any of the three different views was measured and recorded. Posterior wall impact and loss of acetabulum congruity (or femoral head subluxation) were also recorded.

**Fig. 4:**
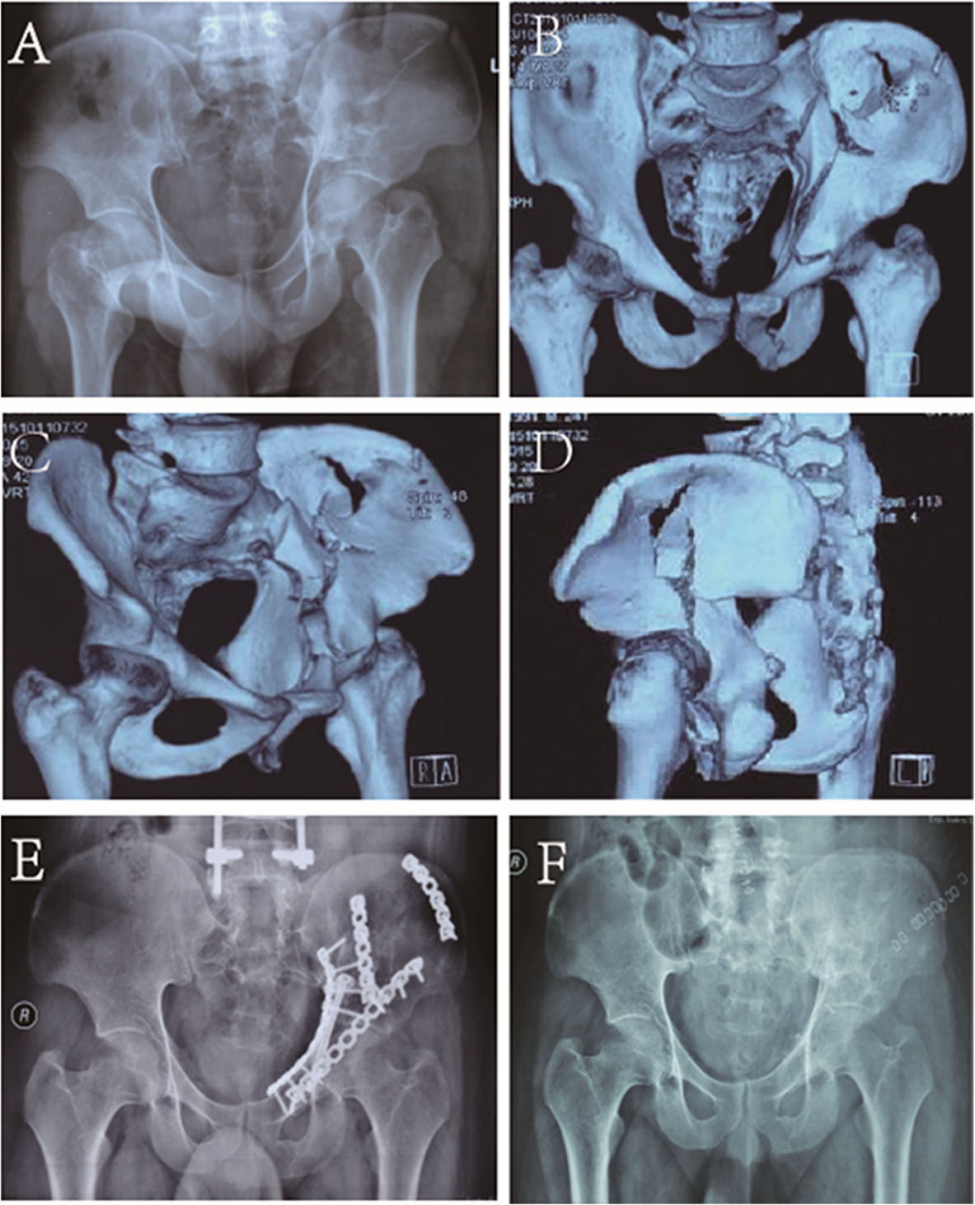
Male, 24 years old, high falling injury, fracture of the anterior column, upper posterior column, pubic and iliac bone. The operation was managed by the supra-ilioinguinal approach. Anteroposterior (AP) position of pre-operation (**a**). Three-dimensional (3D) AP projection and 3D reconstructed Judet views before the operation (**b**–**d**). Anteroposterior position of post-operation (**e**). Two years post-operation, AP view after removing all the internal fixation (**f**)

All patients were regularly followed up postoperatively at the outpatients’ department at an interval of 1 month, 3 months, 6 months and 1 year and then yearly if required (Fig. 4). Follow-up hip function score was graded by the Merle d’Aubigne and Postel score system [17]: excellent (18 points), good (15–17 points), fair (13–14 points) or poor (> 13 points) according to the sum of score of pain, walk and range of motion.

### Statistical analysis

Statistical analysis of the data was performed using SPSS 22.0 (Chicago, IL, USA). Descriptive statistics were expressed as means ± standard deviations for continuous variables, while nominal variables were expressed as number (percentage). The assumptions of normality and homogeneity of variance were tested using the Kolmogorov-Smirnov test. Comparisons between two groups of continuous variables were done by two independent samples Student’s *t* test. Comparisons of categorical data were done by the Pearson Chi-square test or Fisher’s exact test. A value of *P* < 0.05 was considered statistically significant.

## Results

### Surgical details

The mean operative time was shorter and the mean intraoperative haemorrhage was less in group A when compared to that in group B. The difference was statistically significant (operative time *P* = 0.025, intraoperative haemorrhage *P* = 0.003; Table 2). Patients in group A had only one incision whereas 20 (83.3%) patients in group B had an additional iliac window incision. Additional KL approach was utilized in 8 (22.2%) cases in Group A while in Group B, it was used in 7 (29.2%) cases. The difference was not statistically significant (*P* = 0.543) (Table 2).

**Table 2:** Observed indicators of the two groups during the surgery

		Supra-ilioinguinal	Modified Stoppa	Test value	*P* value
Operative time (min)		185.7 ± 38.9	212.5 ± 51.3	2.298	0.025^#^
Intraoperative haemorrhage		801.9 ± 332.4	1087.5 ± 366.3	3.129	0.003^#^
(ml)					
Iliac window		-	20 (83.3%)		^*^
Additional KL		8 (22.2%)	7 (29.2%)	0.370	0.543*
Complications					0.741^**^
DVT		2 (5.6%)	1 (4.2%)		
LFCNI		3 (8.3%)	–		
Fat liquefaction of incision		1 (2.8%)	2 (8.3%)		
Obturator nerve palsy		–	1 (4.2%)		
Vascular injury		–	1 (4.2%)		
Total rate		6 (16.7%)	5 (20.8%)	0.167	0.683^*^

Categorical variables are given as absolute numbers with percentages in parentheses. Noncategorical variables are given as mean ± SD

*KL* Kocher-Langenbeck approach, *DVT* deep vein thrombosis, *LFCNI* lateral femoral cutaneous nerve injury

*Pearson Chi-square test

**Fisher’s exact test

^#^Two independent samples Student’s *t* test

### Radiographic analysis

When measured on radiography, the quality of reduction was graded as anatomical in 22 (61.1%) cases, imperfect in 10 (27.8%) cases and poor in 4 (11.1%) cases in group A according to Matta criteria [2]. While in group B, it was graded as anatomical in 14 (58.3%) cases, imperfect in 7 (29.2%) cases and poor in 3 (12.5%) cases. There was no significant statistical difference between the two groups (*P* = 0.974) (Table 3).

**Table 3:** The imaging and clinical results among the two groups

	Supra-ilioinguinal	Modified Stoppa	Test value	*P* value
CT measurement (mm)				
Mean gap	3.7 (0.5–9.2, 1.7)	3.8 (0.4–10.1, 2.0)	0.213	0.832^#^
Mean step	0.8 (0–4.3, 0.8)	1.0 (0–7.3, 1.5)	0.660	0.512^#^
Radiological outcome (Matta)			0.052	0.974^*^
Anatomical (< 1 mm)	22 (61.1%)	14 (58.3%)		
Imperfect (2–3 mm)	10 (27.8%)	7 (29.2%)		
Poor (> 3 mm)	4 (11.1%)	3 (12.5%)		
Merle D’Aubigne-Postel score			0.238	0.971^*^
Excellent (18)	20 (55.6%)	12 (50.0%)		
Good (15–17)	12 (33.3%)	9 (37.5%)		
Fair (13, 14)	3 (8.3%)	2 (8.3%)		
Poor (< 13)	1 (2.8%)	1 (4.2%)		

Data are presented as means (range, SD) in millimetres of the gaps/steps measurements (CT scan evaluation). The radiological outcome and clinical results are presented as number (percentage)

*Pearson Chi-square test

^#^Two independent samples Student’s *t* test

On postoperative CT, the mean gap displacement in group A was 2.3 mm (range 0.6–9.2 mm, SD 1.4 mm), which was approximative to 2.1 mm (range 0.1–10.1 mm, SD 2.1 mm) required for group B (*P* = 0.689). The mean step displacement (group A 0.8 mm (range 0–4.3 mm, SD 0.8 mm), group B 1.0 mm (range 0–7.3 mm, SD 1.5 mm), *P* = 0.512) did not differ markedly as well (Table 3).

### Perioperative complications and follow-up results

All patients were followed up in the outpatient department and the mean follow-up time of group A was 13.5 months (12–28 months) and for group B was 18.9 months (12–48 months). The complications are presented in Table 2. Two cases of postoperative deep vein thrombosis (DVT) and one case of fat liquefaction of incision were observed in each of the groups. Three lateral femoral cutaneous nerve injury (LFCNI) occurred in group A, and they all recovered in 3 to 12 months after surgery. In group B, obturator nerve palsy occurred in one case which recovered 6 months later. The external iliac vein lacerated in one case during the surgery and it was sutured immediately without thrombosis or hemorrhage occurred after operation. There was no significant difference in the incidence of overall complications between the two groups (*P* = 0.683).

According to the Merle d’Aubigne and Postel score system [17], the clinical outcomes achieved in group A (excellent in 20 (55.6%) cases, good in 12 (33.3%) cases, fair in 3 (8.3%) cases, and poor in 1 (2.8%) cases) were similar to that in group B (excellent in 12 (50.0%) cases, good in 9 (37.5%) cases, fair in 2 (8.3%) cases, and poor in 1 (4.2%) cases) at the last follow-up (*P* = 0.971) (Table 3).

## Discussions

This is the first direct comparison of the surgical features, complications, radiographic and clinical results of the new supra-ilioinguinal approach and the widely used modified Stoppa approach for the surgery of acetabular fractures. In this study, operative time and the intraoperative haemorrhage of the supra-ilioinguinal approach were significantly shorter and less than the modified Stoppa approach. This result could be reasonably elucidated by the exposed clear vision and easy operation of the supra-ilioinguinal approach. The rate of complication, anatomic reduction and hip function recovery by the two approaches had no significant statistical difference. In consideration of the classic and widely used of the modified Stoppa approach [6, 7, 18] for acetabulum fracture, it would be accepted to achieve the similar fracture reduction and function recovery for the supra-ilioinguinal approach.

As with other joint fractures, it is widely recognized that the functional results of the hip and the risk of post-traumatic arthritis after surgical treatment of acetabular fractures are mainly determined by the quality of the reduction and the following solid fixation [19, 20]. Direct buttressing of the quadrilateral plate and the acetabular dome becomes urgent with the increased incidence of the elderly osteoporotic acetabular fractures [21, 22]. The supra-ilioinguinal approach provides a wide visualization from the anterior sacroiliac joint to the pubic symphysis, thus allows direct visualization of the quadrilateral plate [12]. However, there is currently no research data to support the reliability of this approach. It is difficult to compare the effects of different surgical approaches on acetabular fracture reduction, because there is no validated algorithm for the radiographic measurement of gaps and steps to assess the quality of reduction [23]. The Matta criteria is initially used for measurements on postoperative plain radiographs not on CT images. Moreover, none of the previous studies have described the details (where, what and how should be measured) for measuring residual displacement on CT images. To this end, Diederik et al. [16, 24] proposed a standard method, and their study indicates the critical cut-off value to define a poor reduction is greater for gap displacement (5 mm) but smaller for step displacement (1 mm) as assessed on CT. In general, the practice of grading acetabular fracture reduction based on CT measurements is still controversial. But we believe that the residual displacement measured by the standardized method can still reflect the quality of the reduction. On the postoperative CT of groups A and B, the mean gap was 2.3 mm and 2.1 mm, and the mean step was 0.8 mm and 1.0 mm, respectively. There was no significant difference in gap (*P* = 0.689) and step (*P* = 0.512) (Table 3). The results of large series of patients operated by the modified Stoppa approach have been reported by several authors. Sagi et al. [6] described 50 cases of which 92% had an excellent or good reduction of acetabular fractures; Anderson et al. [25] reported an 82% anatomic reduction rate and Shazar et al. [11] described 103 cases of which 82.5% anatomic reduction rate. In our study, satisfactory reduction (anatomical and imperfect reduction) rate was 88.9% in group A, while 87.5% in group B which is comparable to theirs. There was no statistical difference (*P* = 0.974).

There were 8 (22.2%) patients with acetabular fracture who were treated with KL approach in group A (25% were elementary), and 7 (29.2%) patients in group B (25% were elementary) were treated with an additional KL approach (Table 2), with no statistical difference (*P* = 0.543). Clinical results at the last follow-up were evaluated using the Merle D’Aubigne-Postel score system: 88.9% in group A and 87.5% in group B were graded good or excellent (*P* = 971). This is in agreement with results of previous reports [7, 11, 25].

The operative time and intraoperative haemorrhage was shorter and less of the supra-ilioinguinal approach, when the anterior approach was calculated separately, which was similar to that in other studies [11, 12]. This may be due to fewer surgical incision extensions and a more convenient reduction operation (Table 2). The modified Stoppa approach provides improved exposure of the quadrilateral surface and posterior column [6]. However, as this is a midline approach, it does not expose the iliac wing and the anterior wall of the acetabulum and cannot deal with fractures of the anterior 1/3 of the iliac bone and the anterior wall of the acetabulum. Thus, an additional lateral window (20 cases, 83.3%) of the ilioinguinal approach is required for the application of reduction clamps and insertion of posterior column lag screws through a pelvic brim plate in the supra-pectineal position.

The overall incidence of postoperative complications in the two groups (16.7% in group A and 20.8% in group B, *P* = 0.683) was comparable. Increased lateral iliac window may increase complications such as deep infections, although it did not occur in our study. The complication of fat liquefaction of incision was comparable, and neither patient developed a more serious infection. Prophylactic use of antibiotics may play an important role. Another important limitation of the modified Stoppa approach is the remarkable rectus tension which increases traction difficulty and may strangle the iliac vessels and lead to a high risk of injury and thrombotic lesion. In patients who underwent surgery with the modified Stoppa approach, 2 (8.3%) patients had DVT and 1 (4.2%) patient had external iliac vein injury due to excessive traction of the iliac vessels. However, only 2 (5.6%) patients had with DVT in group A. These results stress that the exposure of the iliac vessels may increase awareness of protection and improve management of vascular injury (laceration and thrombotic lesion) [7]. Most articles indicate that nerve injury is one of the frequent postoperative complications of acetabular fracture surgery, with rates ranging from 2 to 26% [6, 26]. The major nerve including femoral and sciatic nerve was not injured in our study, but there was 3 (8.3%) lateral femoral cutaneous nerve injury in group A and 1 (4.2%) obturator nerve palsy in group B. These nerve palsy recovered at 3 to 12 months of follow-up. No inguinal hernias occurred in either group.

The supra-ilioinguinal approach can easily deal with acetabular fractures involving the quadrilateral plate and the anterior wall of the acetabulum without mini-incision at the iliac crest like Keel [13, 14] used in the pararectus approach. Since the incision is quite close to the quadrilateral plate, it is more convenient to reduce and fix the fracture thus reducing intraoperative bleeding and lower the risk of complications like neurovascular injury. Compared to the modified Stoppa/iliac approach, the supra-ilioinguinal approach provides a better exposure of the anterior 1/3 of the iliac bone or sacroiliac joint injuries and enables the optimal downward reduction force to be applied in the reduction of the superior displacement of the anterior column fracture at the pelvic brim. It can be a better choice for patients with a history of lower abdominal disease like caesarean section, bladder injury or surgery. A potential disadvantage of the supra-ilioinguinal approach might be for the treatment of bilateral acetabular fractures whereas it can be operated using a single incision by the modified Stoppa approach. The supra-ilioinguinal approach is contraindicated in fractures with posterior-only patterns. It is not suitable for obese patients or those presenting with abdominal distension, ileus or bowel obstruction.

The main shortcoming of this study is that it is a retrospective study, which makes it lower in evidence level. Randomized prospective study involving a larger number of cases and long-term follow-up are needed to estimate whether the supra-ilioinguinal approach is comparable to or superior to the standard approach in terms of functional outcomes and the incidence of hip osteoarthritis.

## Conclusions

Compared with the modified Stoppa approach, the supra-ilioinguinal approach provides a closer visualization to the quadrilateral plate, the operative time was shorter and the intraoperative haemorrhage was clearly less. It is at least equal to or could be a better choice to deal with complicated acetabular fractures especially involving the quadrilateral plate and the anterior one third of the iliac bone.

## Data Availability

The datasets generated and/or analysed during the current study are available from the corresponding author by reasonable request.

## Abbreviations

3D: Three-dimensional
AIP: Anterior intra-pelvic approach
ASIS: Anterior superior iliac spine
CT: Computerized tomography
DVT: Deep venous thrombosis
KL: Kocher-Langenbeck approach
LFCNI: Lateral femoral cutaneous nerve injury
SD: Standard deviation
